# Retraction statement: Luo L, Liu H and Xi Q (2018) Trastuzumab induces PUMA‐dependent apoptosis and inhibits tumor growth in gastric cancer. *FEBS Open Bio* 8, 1911–1919.

**DOI:** 10.1002/2211-5463.13587

**Published:** 2023-03-17

https://doi.org/10.1002/2211-5463.12522

The above article, published online on 15 September 2018 in Wiley Online Library (wileyonlinelibrary.com), has been retracted by agreement between the Editor‐in‐Chief, FEBS Press, and John Wiley and Sons Ltd. The retraction has been agreed following an investigation into concerns raised by a third party, which revealed inappropriate duplications between this and other published articles [1, 2, 3, 4]. Thus, the editors consider the conclusions of this manuscript substantially compromised.
